# Comparative study of the efficacy and safety of topical minoxidil 2% versus topical bimatoprost 0.01% versus topical bimatoprost 0.03% in treatment of eyebrow hypotrichosis: a randomized controlled trial

**DOI:** 10.1007/s00403-023-02679-2

**Published:** 2023-07-30

**Authors:** Mohamed S. Zaky, Osama A. Hashem, Sara M. Mahfouz, Mohamed L. Elsaie

**Affiliations:** 1 Department of Dermatology, Venereology and Andrology, Damietta Faculty of Medicine, Al-Azhar University, Damietta, Egypt; 2 Department of Dermatology, Venereology and Andrology, Medical Research and Clinical Studies Institute, National Research Centre, Giza, Egypt

**Keywords:** Bimatoprost, Minoxidil, Hypotrichosis

## Abstract

Eyebrows are an important feature of facial identity and communications in human beings as well as an important eye defense shield from dust and foreign bodies. To compare the efficacy and safety between 0.01%, 0.03% bimatoprost and minoxidil 2% in gel formulations for eyebrow enhancement. Sixty eligible subjects were female or male, aged 18 years or older with eyebrow hypotrichosis, defined as either a Grade 1 or 2 on the Global Eyebrow Assessment (GEBA) scale. Patients were randomized into 3 groups using block randomization. Group a (20 patients) applied topical 0.03% bimatoprost gel once daily onto both eyebrows, group b (20 patients) applied topical 0.01% bimatoprost gel once daily onto both eyebrows while group c (20 patients) applied topical minoxidil 2% gel once daily onto both eyebrows. A significant improvement in GEBA score was reported in all the three groups after treatment (*P* ≤ 0.001); however, there was no statistically significant difference between the three groups (P1 = 0.091; P2 = 0.102; P3 = 0.663). Bimatoprost is equally efficacious as minoxidil in enhancement of eyebrows with a more favorable response produced by the 0.03% concentration.

## Introduction

Eyebrows are an important feature of facial identity and communications in human beings as well as an important eye defense shield from dust and foreign bodies [1]. Loss of eyebrow, known as eyebrow hypotrichosis negatively affects an individual's self image and confidence levels and can impact psychosocial interactions [2]. No standard evidence-based treatment exists for eybrow hypotrichosis apart from topical medications, intralesional injections, surgical transplantation as well as camouflage and microblading [3].

The eyebrow hair cycle is comprised no differently from other hairs of three phases (anagen, catagen and telogen) however with specific characteristics. Almost 10–15% of eyebrow follicles are in anagen phases which last from 15 to 30 days in average while the majority of follicles (85–90%) are in telogen phase which last for 60–90 days [4].

Minoxidil has been approved for treating male and female pattern hair loss and was found to stimulate hair growth by limiting and regressing the telogen phase of hair follicles as well as by prolongation of the anagen phase resulting in increased hair follicle size. A few number of reports and studies proved an eyebrow enhancement and favorable safety effect of topical minoxidil use in eyebrow hyptrichosis [5].

Bimatoprost is a prostamide, a sytnthetic prostaglandin used to treat glaucoma. The mechanism of bimatoprost and prostamides for enhancing hair growth is still unclear. It is well-known that prostaglandin receptors exist in dermal hair papillae and outer root sheath of the hair follicles. It is predicted that bimatoprost may stimulate the transition of hair follicles from the telogen to the anagen phase and prolong the duration of the anagen phase as well as stimulate melanogensis increasing hair length and darkness [6].

Bimatoprost in its 0.03 and 0.01 formulations was studied in a number of recent studies and compared to one another which revealed its safety and efficacy as a therapeutic potential for eyebrow hypotrichosis [7–10].

To our knowledge, there has been no previous report of randomized trials comparing, the efficacy and safety between 0.01%, 0.03% bimatoprost and minoxidil 2% in gel formulations for eyebrow enhancement has been published to date which is the main aim of the presented study.

## Patients and methods

This was a randomized, triple armed single blinded randomized study to compare the efficacy and safety of topical 0.01% and 0.03% bimatoprost versus topical 2% minoxidil. The sample size was calculated based on data from a previous comparative study in a Thai population, with a 5% significance level and 90% power. The calculated minimum number of subjects was 50. Considering dropout, we recruited a total of 60 subjects. This study was approved by the ethical committee for research in AL-Azhar University, Damietta faculty of medicine and all patients provided written informed consent prior to starting the study.

Sixty eligible subjects were female or male, aged 18 years or older with eyebrow hypotrichosis, defined as either a Grade 1 or 2 on the Global Eyebrow Assessment (GEBA) scale. The GEBA instrument is a validated 4-point scale used to grade fullness of the eyebrows (1 = very sparse, 2 = sparse, 3 = full, and 4 = very full). Subjects with recent eye operations, those suffering from systemic diseases that can possibly affect eyebrow loss; alopecia areata, trichotillomania, thyroid diseases, atopic dermatitis, seborrheic dermatitis; and those receiving treatment of hypotrichosis within 6 months of enrollment were excluded. Females who had microblading, eyebrow tattoos at screening and female subjects who were pregnant, nursing, or planning for a pregnancy during the study were excluded.

Patients were randomized into 3 groups using block randomization. Group a (20 patients) applied topical 0.03% bimatoprost gel once daily onto both eyebrows, group b (20 patients) applied topical 0.01% bimatoprost gel once daily onto both eyebrows while group c (20 patients) applied topical minoxidil 2% gel once daily onto both eyebrows. Containers were identical and were different in color for each. Each bottle was labeled with a container number, dosing instruction, and storage condition. Containers were provided to subjects and refilled at every scheduled follow up monthly visit.

The study was scheduled for 16 weeks and at every visit standard photographs were taken using digital camera and trichoscopic eyebrow assessment was carried out using DL-4.

Hair count and diameter were assessed using a folliscope and measurements were performed up a vertical line drawn from mid papillary line. Eyebrow number and diameter were analyzed using image analysis software and by manual count.

For outcomes, the GEBA scale was evaluated from standard photographs by two blinded dermatologists as subjective parameter in every visit. Another outcome measurement in our study was the 7-point rating scale, defined as follows: marked deterioration (− 3), moderate deterioration (− 2), slight deterioration (− 1), no change (0), slight improvement (+ 1), moderate improvement (+ 2), and marked improvement (+ 3).

### Preparation of minoxidil 2% gel (20 mg/g)

The first step was to dissolve 1% carbopol in distilled water (2 g is dissolved in 100 ml of distilled water under stirring conditions), the second step was to activate carbopol gel formation by using seven drops of triethanolamine for every 100 ml of gel, and the third step was to incorporate the gel preparation into the base with 5 min of continuous stirring and stirring to obtain a homogeneous clear drug–gel solution.

### Preparation of bimatoprost gel

The first step was to dissolve 1% carbopol in distilled water (2 g is dissolved in 100 ml of distilled water under stirring conditions), the second step was to activate carbopol gel formation by using 0.01 gm or 0.03 gm of bimatoprost for every 100 ml of gel, and the third step was to incorporate the gel preparation into the base with 5 min of continuous stirring and stirring to obtain a homogeneous clear drug–gel solution.

## Results

Sixty patients; all females were included and completed the study till the end. The mean age for bimatoprost 0.03% group was (34.40 ± 12.04) while bimatoprost 0.01% group mean age was (36.10 ± 11.22) and minoxidil 2% group mean age was (32.30 ± 11.66). There was no significant difference between the studied groups regarding age and sex.

**Fig. 1:**
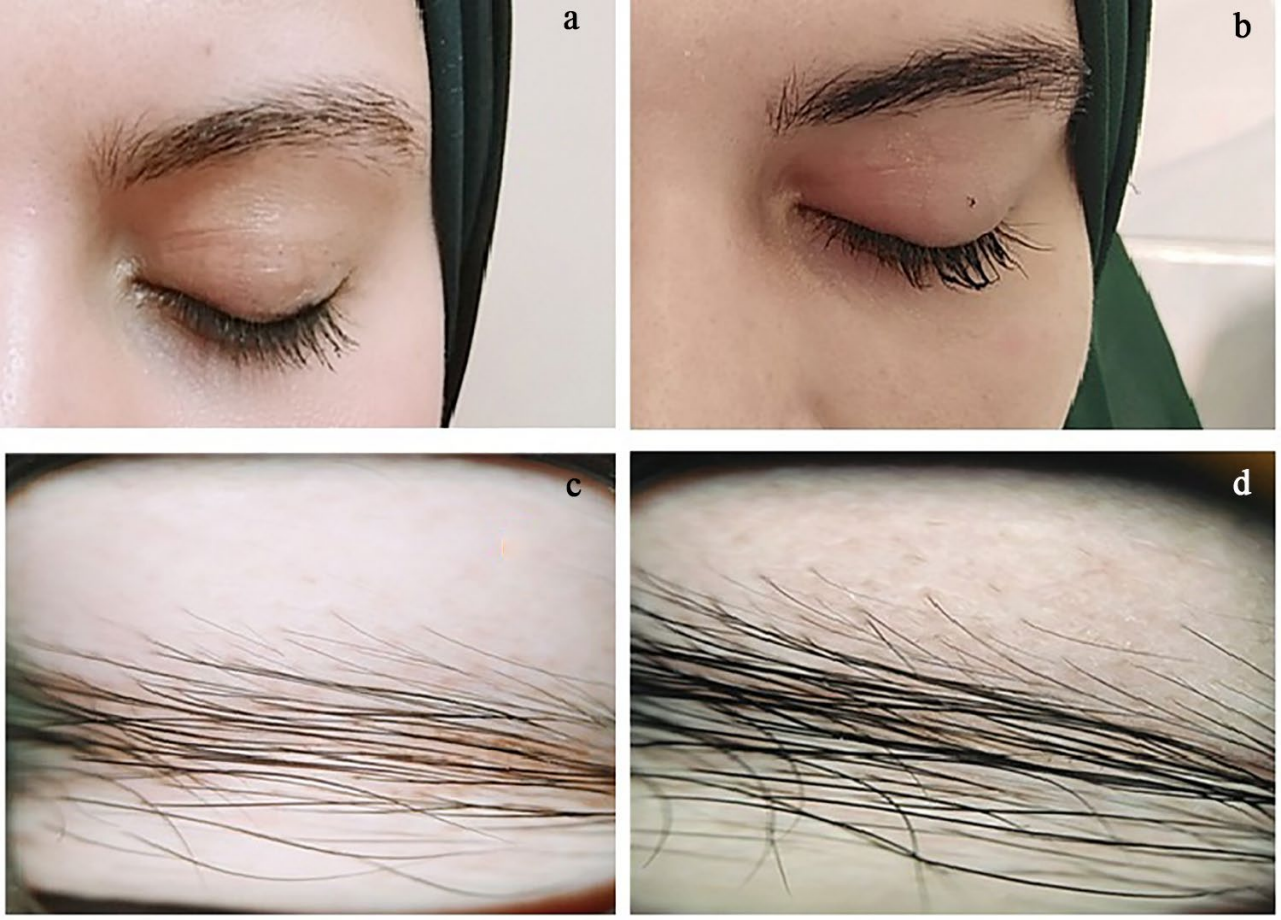
Clinical and dermoscopic image of a case of primary eyebrow hypotrichosis before (**a**, **c**) and 16 weeks after treatment with topical bimatoprost 0.03% (**b**, **d**)

**Fig. 2:**
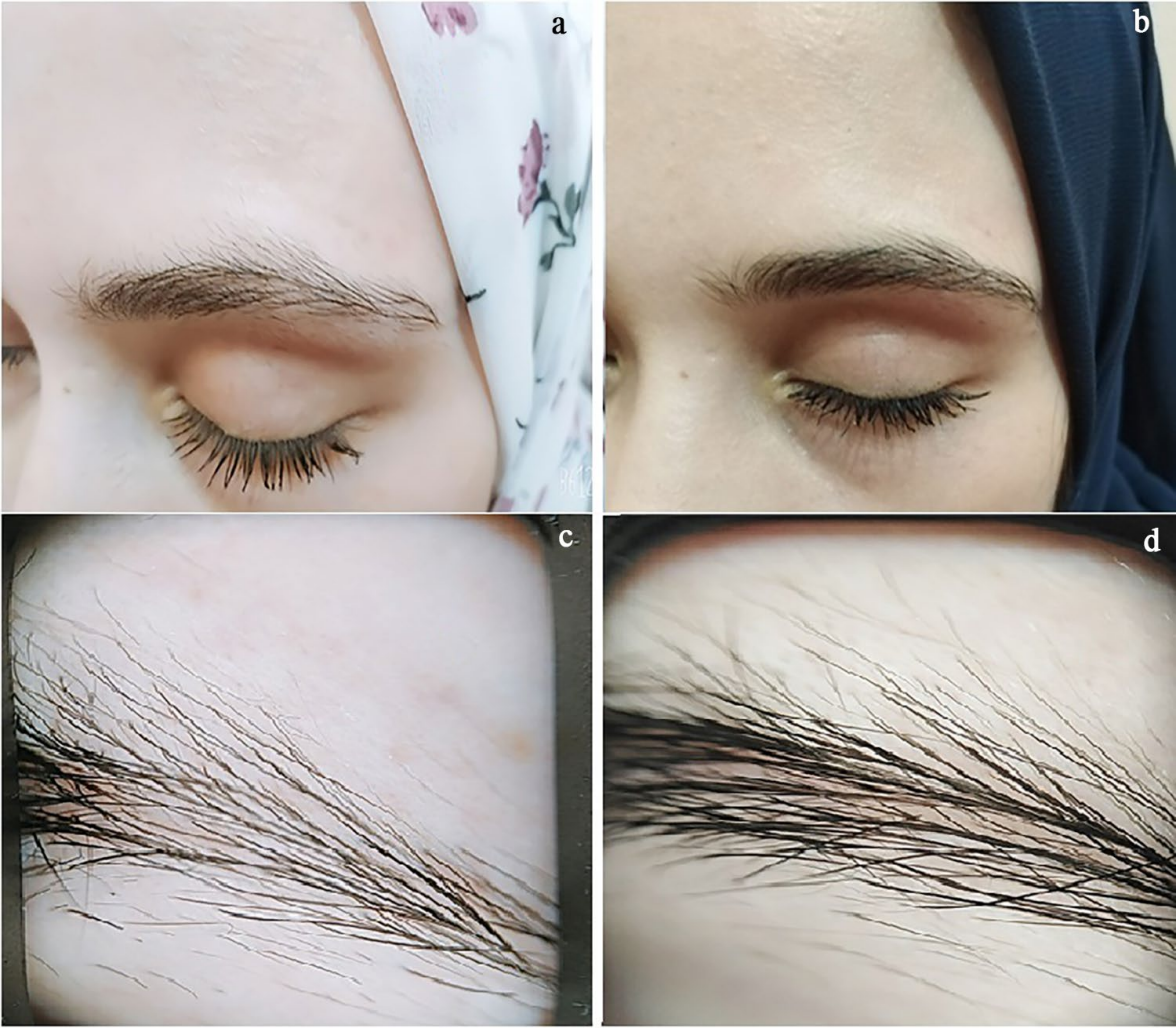
Clinical and dermoscopic image of a case of primary eyebrow hypotrichosis before (**a**, **c**) and 16 weeks after treatment with topical bimatoprost 0.01% (**b**, **d**)

**Fig. 3:**
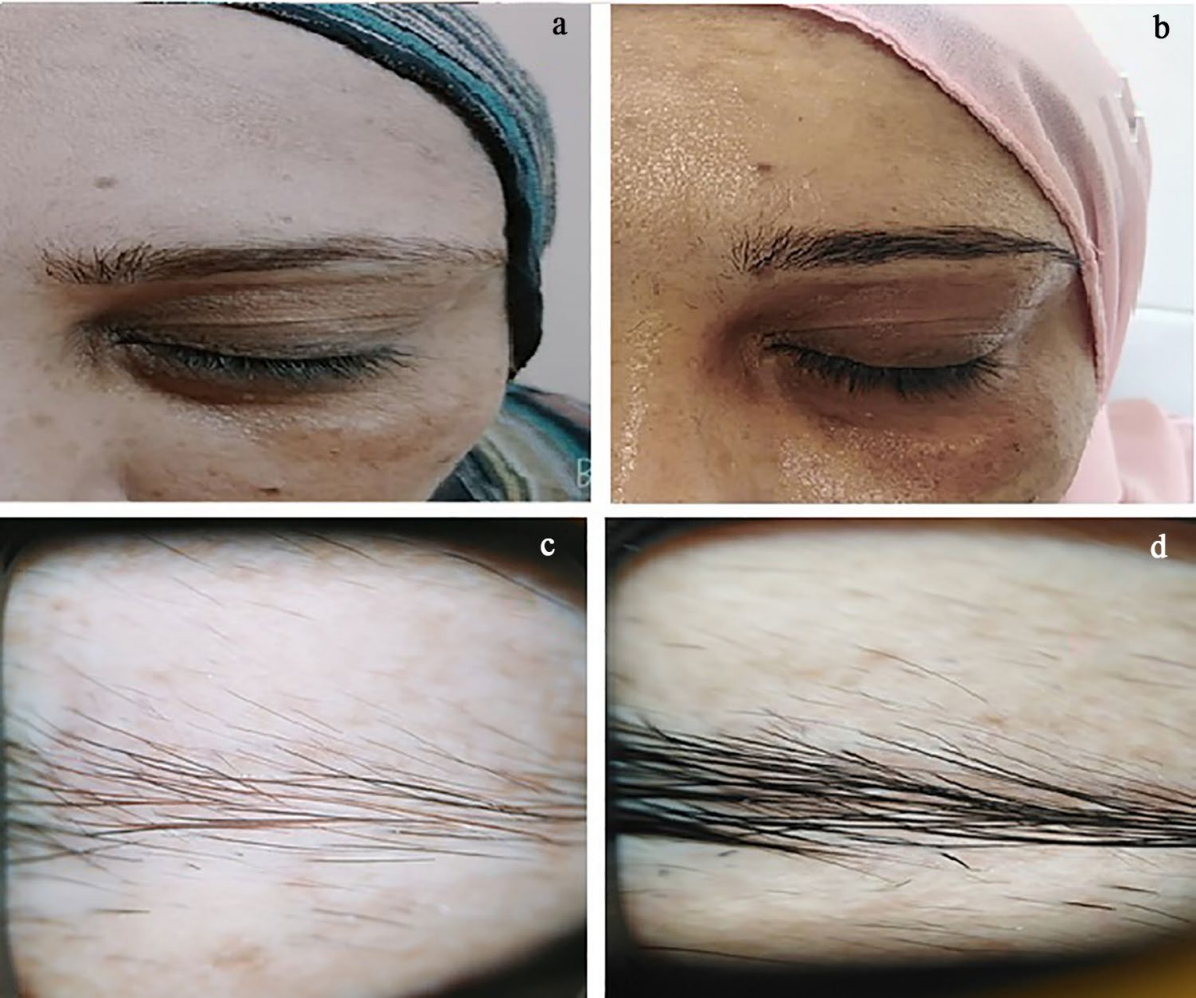
Clinical and dermoscopic image of a case of primary eyebrow hypotrichosis before (**a**, **c**) and 16 weeks after treatment with topical minoxidil 2% (**b**, **d**)

Clinical assessment of the GEBA scale prior to the study revealed no significance among the included subjects. After 4 months of treatment, the proportion of patients with at least a 1-grade improvement in GEBA scale was 100% in the 0.03% bimatoprost group compared to 80% of patients in both of the bimatoprost 0.01% and minoxidil 2% groups. A significant improvement in GEBA score was reported in all the three groups after treatment (*P* ≤ 0.001); however, there was no statistically significant difference between the three groups (P1 = 0.091; P2 = 0.102; P3 = 0.663). Table 1This was in accordance with the investigators 7 point rating scale that revealed a significant improvement in all three groups (*P* ≤ 0.05); which was more favorable in the bimatoprost 0.03% group Table 2; Figs. 1, 2, and 3.

**Table 1:** Improvement in global eyebrow assessment scale before and after treatment

GEBA	Bimatoprost 0.03% Group, *n* (%)	Bimatoprost 0.01% Group, *n* (%)	Minoxidil 2% Group, *n* (%)	Test of significance	Within group significance
Before					P1 = 0.057
Very sparse	12 (60.0)	6 (30.0)	6 (30.0)	*χ*^2^ = 5.0	P2 = 1.0
Sparse	8 (40.0)	14 (70.0)	14 (70.0)	*P* = 0.082	P3 = 0.06
After				MC *P* = 0.251	P1 = 0.091 P2 = 0.102 P3 = 0.663
Sparse	0 (0.0)	4 (20.0)	4 (20.0)		
Full	16 (80.0)	14 (70.0)	12 (60.0)		
Very full	4 (20.0)	2 (10.0)	4 (20.0)		
Comparison between before and after treatment	SM = 4.36 *P* < 0.001*	SM = 4.24 *P* < 0.001*	SM = 4.24 *P* < 0.001*		

GEBA, Global eyebrow assessment scale; *χ*^2^ = Chi-Square test MC:Monte Carlo test, SM:Stewart Maxwell test, *P*: probability, P1: difference between Bimatoprost.03 and Bimatoprost 0.01%, P2: difference between Bimatoprost.03 and minoxidil2%, P3: Bimatoprost.01% and minoxidil2%, *Statistically significant if *P* < 0.05

**Table 2:** Clinical improvement by 7-point rating scale

Rating scale	Bimatoprost 0.03%group, *n* (%)	Bimatoprost 0.01% group, *n* (%)	Minoxidil 2% group, *n* (%)	Test of significance	Within group significance
1	3 (15.0%)	8 (40.0%)	6 (30.0%)	MC *P* = 0.375	P1 = 0.123 P2 = 0.449 P3 = 0.710
2	8 (40.0%)	8 (40.0%)	8 (40.0%)		
3	9 (45.0%)	4 (20.0%)	6 (30.0%)		
Average Rating score	2.3	1.8	2		

Clinical improvement by 7-point rating scale comparing both concentrations by blinded evaluators; rating scale; 1 = slightly improvement, 2 = moderately improvement, 3 = marked improvement, n (%): number, percentage, MC:Monte Carlo test, p:probability, P1: difference between Bimatoprost.03 and Bimatoprost 0.01%, P2: difference between Bimatoprost.03 and minoxidil2%, P3: Bimatoprost.01% and minoxidil2%, *Statistically significant if p < 0.05

At 16 weeks of treatment, a statistically significant change in hair diameter was observed in all 3 groups. Moreover, the eyebrow hair count increased significantly from baseline in all three groups. Hair diameter and number were superiorly but none significantly increased in the bimatoprost 0.03 group when compared to the other two groups Table 3.

**Table 3:** Mean changes from baseline in eyebrow diameter and number at week 16

	Mean change from baseline (mean ± SE)			Difference between groups
	Bimatoprost 0.03%	Bimatoprost 0.01%	Minoxidil 2%	
Diameter (μm)	4.23 ± 1.41 (*P* = 0.001)	4.07 ± 1.02 (*P* = 0.001)	3.98 ± 3.18 (*P* < 0.007)	P1 = 0.123 P2 = 0.449 P3 = 0.710
Number	5.97 ± 5.67 (*P* < 0.001)	5.45 ± 3.97 (*P* < 0.001)	4.96 ± 3.24 (*P* < 0.001)	P1 = 0.091 P2 = 0.102 P3 = 0.663

*P*: probability, P1: difference between Bimatoprost 0.03 and Bimatoprost 0.01%, P2: difference between Bimatoprost 0.03 and minoxidil2%, P3: difference between Bimatoprost 0.01% and minoxidil 2%, *Statistically significant if *P* < 0.05

At the end of the study, 100% of patients in all three groups reported improvement in their eyebrow appearance on the self-evaluation with 7-point rating scale. Moreover, using a satisfaction rating scale ranging from 1 to 10; patients demonstrated a significantly higher satisfaction score of (8.0 ± 0.65) with bimatoprost 0.03% when compared to bimatorpost 0.01% (6.80 ± 0.62) and minoxidil 2% (6.60 ± 0.50) respectively (p1 ≤ 0.01; p2 ≤ 0.01) Table 4.

**Table 4:** Comparison of satisfaction scale between studied groups

Satisfaction scale	Bimatoprost 0.03% Group, *n* (%)	Bimatoprost 0.01% group, *n* (%)	Minoxidil 2% Group, *n* (%)	test of significance	Within group significance
6	0	6(30.0)	8(40.0)	MC *P* < 0.001*	P1 < 0.001* P2 < 0.001* P3 = 0.319
7	4(20.0)	12(60.0)	12(60.0)		
8	12(60.0)	2(10.0)	0		
9	4(20.0)	0	0		
Mean ± SD	8.0 ± 0.65	6.80 ± 0.62	6.60 ± 0.50	*F* = 32.68 *P* < 0.001*	P1 < 0.001* P2 < 0.001* P3 = 0.290

Patient satisfaction score using a visual analog scale, ranging from “0” (least satisfied) to “10” (most satisfied). MC:Monte Carlo test, F: F:One Way ANOVA test, *P*: probability, P1: difference between Bimatoprost.03 and Bimatoprost 0.01%, P2: difference between Bimatoprost.03 and minoxidil2%, P3: Bimatoprost.01% and minoxidil2%, *Statistically significant if *P* < 0.05

Safety evaluation in terms of adverse events were negligible and of no statistical significance among all groups. A single case of contact dermatitis was reported by a minoxidil user while two cases complained of pruritis and mild erythema that resolved spontaneously without any complaints.

## Discussion

In the current study a significant improvement in GEBA score was reported in all the three groups after treatment (*P* ≤ 0.001); however, there was no statistically significant difference between the three groups (P1 = 0.091; P2 = 0.102; P3 = 0.663). At 16 weeks of treatment, a statistically significant change in hair diameter was observed in all 3 groups. Moreover, the eyebrow hair count increased significantly from baseline in all three groups.

Bimatoprost use for eyebrow hypotrichosis remains empirical and the exact mechanism of action on eyebrow growth remains to be further elucidated. Animal studies revealed that bimatoprost can prolong the anagen duration and further prolongs telogen to anegen transition phase. This results in increased hair growth and length. Furthermore, bimatoprost had been shown in mouse models to increase the hair bulb and papillae diameters as well as increase melanogenesis which results in increased hair thickness and pigmentation [11, 12].

In 2001, hypertrichosis was reported in 77% of glauacoma patients using latanoprost [13]. Furthermore eye lash lengths of children and adults using latanoprost ophthalmic solution for glaucoma was found to be increased [14].

Eyelash growth was also reported in 12.6 to 35.7% of patients using bimatoprost 0.03% and was even more superior to latanoprost when compared with [15–17]. The efficacy of bimatoprost 0.03% for the treatment of eyelash hypotrichosis was proven by many randomized, controlled trials and was approved by the FDA in 2008 [18].

This three armed randomized trial represents the first of its nature to compare the safety and efficacy of bimatoprost in its 0.03% and 0.01% concentrations as well as minoxidil 2% topical gel. A significant improvement was demonstrated in all three groups; with a more non-significantly favorable response demonstrated by the bimatoprost 0.03% group.

Earlier case reports demonstrated good response to eyebrow thinning with the use of bimatoprost 0.03% for 16 weeks and with no side effects reported [8, 9]. Two randomized trials compared the efficacy of bimatoprost 0.03% to either minoxidil 3% or a vehicle solution for 4 months and 9 months, respectively, and reported an equipotent response of bimatoprost to minoxidil while a superior response of bimatoprost to vehicle was demonstrated [7, 19].

Vergilis–Kalner in their pilot study showed a significant eyebrow growth using bimatoprost 0.03% for 6 weeks when compared to placebo. All ten (10) subjects included demonstrated eyebrow thickening and growth with no side effects [20].

Bimatoprost 0.03% twice daily use versus single daily use versus placebo was compared in a randomized trial for patients complaining of eyebrow thinning. The proportion of subjects with improvement was significantly higher and comparable in both bimatoprost groups versus vehicle (both, *P* < 0.001) [21].

In their recent randomized double-blinded split face trial, Suchonwanit et al. [22] compared application of bimatoprost 0.03% versus bimatoprost 0.01% in 30 patients with eyebrow hypotichosis. Both 0.01% and 0.03% bimatoprost significantly improved eyebrow density and diameter (*P* < 0.05) with non-significant superiority of the 0.03% to the 0.01% concentration. In accordance with their results, we demonstrated a non-significant superior response to the 0.03% bimatoprost over 16 weeks of application.

Topical minoxidil 2% solution effectiveness for eyebrow hypotrichosis was evaluated in a first randomized trial by Lee et al. and demonstrated a significant and safe response when compared to placebo [23]. This was confirmed by a randomized, double-blind, placebo-controlled, split-face comparative that demonstrated tolerance and effectiveness of minoxidil 1% lotion in eyebrow hypotrichosis when compared to placebo [24].

Among side effects reported with the use of bimatoprost solution for ophthalmic conditions and eyelash hypotrichosis; conjuctival hyperemia, eye irritation, iris pigmentation, periorbital pigmentation and fat atrophy were the most frequently reported [25]. Unlike with ophthalmic conditions, eyebrow use of bimatoprost was associated with infrequent complaints of skin irritation, pruritis and slight skin pigmentation that resolved on discontinuation of use. In the current study very minimal side effects were reported.

The study was limited by the relatively small sample size; only female sex subjects, the short study and follow-up durations as well as failure of correlating hypotrichosis duration to treatment response.

In conclusion, we demonstrated that bimatoprost is equally efficacious as minoxidil in enhancement of eyebrows. A significant improvement was demonstrated in all three groups; with a more insignificantly favorable response demonstrated by the bimatoprost 0.03% group. Larger multicenter randomized trials with longer duration are warranted for establishing a safe efficacious profiling of the used formulations in eyebrow hypotrichosis.

## Data Availability

The data that support the findings of this study are available from the corresponding author upon reasonable request.
